# Biological Composition of Respirable Particulate Matter in an Industrial Vicinity in South Africa

**DOI:** 10.3390/ijerph16040629

**Published:** 2019-02-21

**Authors:** Oyewale Mayowa Morakinyo, Matlou Ingrid Mokgobu, Murembiwa Stanley Mukhola, Tshifhiwa Godobedzha

**Affiliations:** 1 Department of Environmental Health, Faculty of Science, Tshwane University of Technology, Private Bag X680, Pretoria 0001, South Africa; mokgobumi@tut.ac.za (M.I.M.); mukholams@tut.ac.za (M.S.M.); 2 Department of Environmental Health Sciences, Faculty of Public Health, College of Medicine, University of Ibadan, Ibadan 200284, Nigeria; 3 Air Quality Management, Environment and Agriculture Management Department, City of Tshwane Municipality Private Bag 440, Pretoria 0001, South Africa; tshifhiwaG@tshwane.gov.za

**Keywords:** particulate matter, bioaerosols, air quality, dose rate, health effects, South Africa

## Abstract

There is a growing concern that exposure to particulate matter of aerodynamic diameter of less than 2.5 µm (PM_2.5_) with biological composition (bioaerosols) may play a key role in the prevalence of adverse health outcomes in humans. This study determined the bacterial and fungal concentrations in PM_2.5_ and their inhalation health risks in an industrial vicinity in South Africa. Samples of PM_2.5_ collected on a 47-mm glass fiber filter during winter and summer months were analysed for bacterial and fungal content using standard methods. The health risks from inhalation of bioaerosols were done by estimating the age-specific dose rate. The concentration of bacteria (168–378 CFU/m^3^) was higher than fungi (58–155 CFU/m^3^). Bacterial and fungal concentrations in PM_2.5_ were lower in winter than in the summer season. Bacteria identified in summer were similar to those identified in winter: *Staphylococcus* sp., *Bacillus* sp., *Micrococcus* sp., *Flavobacterium* sp., *Klebsiella* sp. and *Pseudomonas* sp. Moreover, the fungal floras identified include *Cladosporium* spp*., Aspergillus* spp*., Penicillium* spp., *Fusarium* spp. and *Alternaria* spp. Children inhaled a higher dose of bacterial and fungal aerosols than adults. Bacteria and fungi are part of the bioaerosol components of PM_2.5._ Bioaerosol exposure may present additional health risks for children.

## 1. Introduction

Urban air quality in most countries in sub-Saharan Africa is quickly deteriorating mainly due to rapid industrial and population growth [1]. South Africa is one of the largest industrialized economies in the Southern Hemisphere with significant mining and metallurgical activities [2]. It is also an energy and carbon-intensive economy with abundant coal reserves valued at 4% of the world’s total deposits [3]. It is an arid country with high naturally-occurring dust levels, coupled with emissions from industrial and vehicular pollution [4].

South Africa often experiences high air pollution levels that are injurious to human health, mainly in large industrial areas such as the South Durban Industrial Basin and the Vaal Triangle [5]. The Highveld Plateau region accounts for 75% of industrial facilities and is responsible for about 90% of planned emissions of industrial dust, nitrogen oxides and sulphur dioxide in the country [6]. The total estimated annual emission of particulate matter (PM) in the region is 279,630 tons. The emission of PM arising from metallurgical industries accounts for 17% of the total emission. The most commonly reported air pollutants in South Africa include PM, CO, oxides of nitrogen (NO_x)_, SO_2_, O_3_ and Pb [7].

In South Africa, the standard for PM_2.5_ was established in the year 2012 in section 9(1) of the National Environmental Management Act (NEMA): Air Quality Act (AQA) as 40 µg/m^3^ and 20 µg/m^3^ per day and annual average respectively [8]. However, there is no existing legislation governing microbiological standards for air pollution in South Africa. The need to conduct research that will determine the required bio-pollutant levels in PM_2.5_ is of public health importance.

PM with an aerodynamic diameter less than 2.5 microns (PM_2.5_) has garnered wide consideration in recent years and has elicited a wide range of biological responses. PM_2.5_ originates mainly from anthropogenic activities involving vehicular emissions, ground fossil fuel combustion, burning of biomass fuel, construction work, quarrying and mining, agriculture and dust from roads [9]. Owing to its small size, large surface area, penetration ability, deposition, bioavailability and long residence time in the air, PM_2.5_ can penetrate the human respiratory tract [10]. This makes PM_2.5_ more toxic to humans than other common air pollutants [11]. Epidemiological studies [12,13] and toxicological studies [9,14] have associated short- and long-term exposure to PM_2.5_ with a range of adverse health endpoints from acute respiratory infections to untimely deaths. PM_2.5_ is a recognized group 1 carcinogen by the World Health Organization (WHO) and the International Agency for Research on Cancer (IARC) [15]. New evidence suggests possible associations between long-term PM_2.5_ exposure and neurodevelopment, cognitive function and chronic disease conditions such as diabetes in humans [16].

PM_2.5_ is a heterogeneous mix of solid and liquid particles including chemical and biological fractions [17]. A substantial component of PM_2.5_ in indoor and outdoor environments are bioaerosols [18]. Bioaerosols are solid or liquid particles carrying living organisms from biological sources, with sizes ranging from 0.1 mm to 100 mm in diameter [19,20]. They include fungi, bacteria, viruses, endotoxin and pollens that originate from terrestrial and marine environments during biological processes [21]. Bioaerosols are present in the atmosphere as individual organisms, or are attached to PM, dust or water droplets [22]. About 5–10% of total atmospheric particles are suspended PM and about 24% are made up of bioaerosols [23]. 

Bioaerosols vary in size and structure and their diversity is dependent on their source and other prevailing environmental conditions [24]. Bioaerosols get attached to PM to derive their nutrients and be shielded from ultraviolet radiation [25]. Bioaerosols can alter atmospheric chemistry and nucleation processes and interact with ecosystems and human health [26].

The presence of bioaerosols in the air undoubtedly poses a health risk. Researchers have indicated that both viable and non-viable airborne bioaerosols have the potential to cause or aggravate health problems in exposed individuals [27,28]. Health outcomes associated with exposure to bioaerosols have been documented in many studies. For instance, bioaerosols have been implicated in the causality of some non-infectious airway diseases such as hypersensitivity pneumonitis [28], organic dust syndrome, allergies, asthma and rhinitis [29,30,31,32]. Exposure to bioaerosols can trigger exacerbation of asthma and wheeze in both children and adults [33,34]. Bioaerosols have been linked to the impairment of lung function [35,36] and the pathogenesis of pulmonary diseases [37,38], such as chronic obstructive pulmonary disease [39] and severe lung damage [40]. The WHO reported that respiratory tract infections are the foremost cause of death in low-income countries and the 4th leading cause of death in the middle and high-income countries [41].

In South Africa, limited studies have determined the levels of PM_2.5_ originating from an industrial area and the possible risks that could occur from human exposure to its biological contents. There is a growing concern that exposure to microbial bioaerosols may play a key role in the adverse health outcomes in humans [18]. Moreover, there is a dearth of studies in South Africa on the health-related outcomes after exposure to PM_2.5_-bound bioaerosols. A better understanding of the biological component of PM_2.5_ is crucial to bridging the knowledge gap in air pollution and its associated health effects. Therefore, this study determined the bacterial and fungal concentrations in PM_2.5_ in an industrial area in Pretoria West, South Africa. 

## 2. Materials and Methods

### 2.1. Description of Study Area

**Figure 1:**
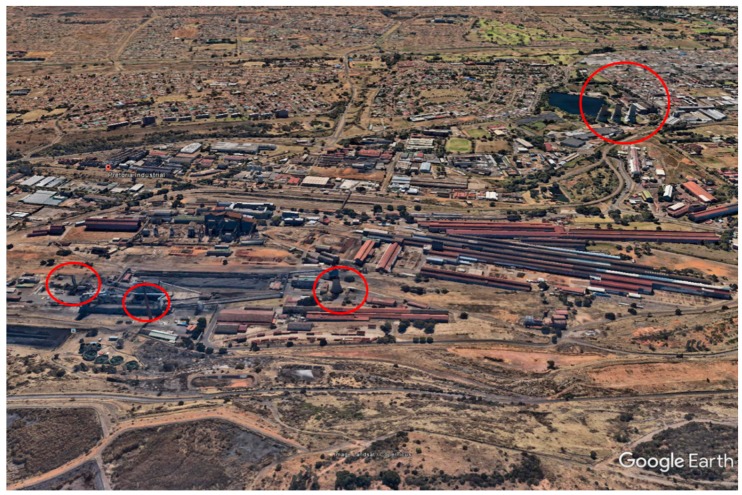
Google Earth image depicting Pretoria West industrial area. The red rings indicate some of the emission stacks in the study area.

The PM_2.5_ samples were collected during winter and summer seasons in an industrial area in Pretoria West, South Africa located at 25°44′46″S 28°11′17″E (Figure 1). 

Pretoria is situated in the northern part of Gauteng province in the North-Northeast of Johannesburg at an altitude of about 1339 m (4393 ft) above sea level [42]. Pretoria is characterized by a humid subtropical climate of long hot rainy summers and short cool to cold, dry winters. The average annual temperature is 18.7 °C (65.7 °F) [24]. The detailed description of the study area was reported in previous studies, such as Morakinyo et al. [43,44].

### 2.2. Sample Collection

**Figure 2:**
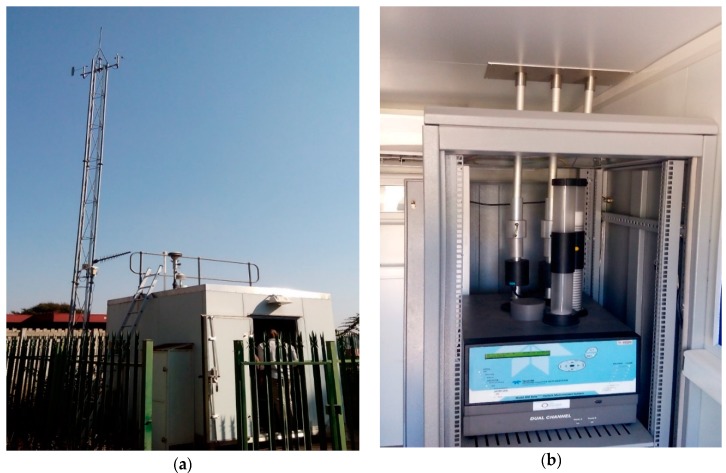
(**a**) A monitoring station in Pretoria West; (**b**) Beta^PLUS^ Particle measurement system.

PM_2.5_ samples were collected on 47-mm quartz fiber filters (with a porosity of 2 µm) using the Beta^PLUS^ Particle measurement system (Figure 2) at a flow rate of 12 L/min for 24 h. The volume of air sampled was normalized to the area of the filter extracted. In the Beta^PLUS^ Particle measurement system, filters move from a supply magazine to the sampling position in succession and finally to the storage magazine for retrieval. The monitoring system is a part of the Air Quality Monitoring network of the City of Tshwane at the Pretoria West industrial area and managed by the Environmental Management Services Department. The equipment actively samples PM_2.5_ in an hourly mode for approximately 57 min. Prior to ambient air sampling, each filter was pre-conditioned for 48 h in a desiccator before and after sampling in a temperature and relative humidity-controlled room (T = 20 ± 1 °C, RH = 50 ± 5%).

The PM_2.5_ samples were collected from 2 January 2016 to 29 February 2016 (summer) and from 1 June 2016 to 31 July 2016 (winter). Overall, 144 filters in four months (i.e., nine filters per week) were used for analysis of the biological content of PM_2.5_. After sampling was completed, filters were retrieved from the Beta^PLUS^ Particle measurement system and placed in separate Petri dishes. The filters’ gravimetric masses were thereafter estimated using a Sartorius ME5-OCE analytical microbalance according to the European Standard EN 14907 [45]. The difference in filter weight, as well as the volume of air that filtered through each filter, were documented.

### 2.3. Filter Analysis

One-half of each of the quartz filters containing the PM_2.5_ samples was dissolved in 50 mL phosphate buffered solution with 0.05% tween 80 (*w/v*), and the mixture was shaken for about one hour. Serial dilutions (10^−3^) were prepared in triplicate [46], and 0.5 mL of each serial dilution was added to Petri plates containing sterile trypticase soy agar for the cultivation of bacteria, and to Petri plates containing sterile malt extract agar for the cultivation of fungi. Bacteria and fungi have been recognized as the primary constituents of PM_2.5_ [47]. They are the most important bioaerosols spores found in outdoor air [47].

The trypticase soy agar (Sigma-Aldrich, St. Louis, MO, USA) and the malt extract agar (Sigma-Aldrich, St. Louis, MO, USA) were supplemented with 50 ppm of cycloheximide and chloramphenicol respectively to prevent the growth of contaminants. The bacterial aerosols on trypticase soy agar were incubated at 28 °C for three days while the fungal aerosols on the malt extract agar were incubated at 28 °C for seven days. Colonies that grew on the media were counted and the mean count was estimated. 

The concentration of culturable bacteria and fungi were computed as colony forming units per cubic meter of air (CFU/m^3^) [48]. The total concentration of cultured bacteria and fungi was computed from the division of the number of colonies counted on the plates by the volume of air sampled [29]. Identification of the bacteria was by morphology and Gram staining [49] while fungal isolates were identified based on the observation of micro- and macro-morphological features [46]. Feller’s law was used as the correction factor [50] while the quantification limit was set at 10 CFU per plate [51].

In ensuring quality control and minimizing errors, characterisation of bioaerosols in PM_2.5_ was done according to the PN-EN 12322 standard [52]. Sterility was achieved by incubating culture medium at an optimal temperature for the procedure for at least 72 h [49]. Blank filters were carried to the field and loaded into the sampling filter holders as were the filters for sampling, but these blank filters received no air flow from the sampler [53]. 

### 2.4. Dose Rate Estimation

The dose rate of the bacterial and fungal component associated with PM_2.5_ was estimated using the United States Environmental Protection Agency (US EPA) model (Equation (1)). The model was developed to assess the risks of environmental exposure for susceptible populations [49,54,55].

(1) $$Dose\;rate\;\left(\mathrm{CFU}/\mathrm{kg}\right)\;=\frac{C\;*\;InhR\;*\;ET}{BW}$$

where:

- C is the bacterial and fungal aerosol concentration (CFU/m^3^);
- InhR is the inhalation rate (m^3^/day);
- ET is the exposure time (h/day);
- BW is the body weight (kg).

The values used for computing these parameters are presented in Table 1. The dose rate was calculated using the US EPA’s Child-Specific Exposure Factors Handbook [56] and other literature [57,58]. The estimation was age specific and divides the population in the study area into four age-specific groups, namely infants (birth–1 year), children (2–5 years), toddlers (6–12 years) and adults (19–75 years). 

### 2.5. Data Analysis

Graphical representation (wind rose) of the effects of wind speed and wind direction on the concentration of PM_2.5_ was done using R^©^ (v.2.13.1) statistical software (Bell Laboratories, Murray Hill, NJ, USA). This was used to deduce the dominant prevailing winds in the study area. Descriptive statistics such as mean, standard deviation and percentages were used to summarize the concentration of bacterial and fungal bioaerosols.

## 3. Results and Discussion

### 3.1. Frequency of Wind Speed and Direction

**Figure 3:**
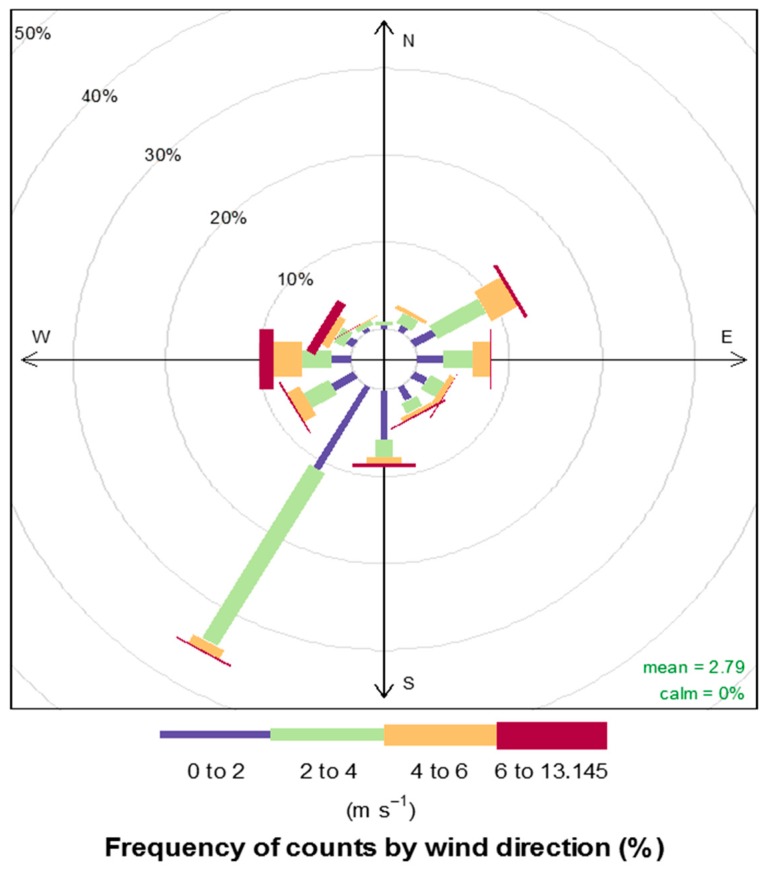
Annual wind rose of wind speed and wind direction in Pretoria West.

The annual hourly frequency spread of wind speed and direction, spanning the entire period of monitoring, is represented by the wind rose diagram (Figure 3). The concentric circles and the radial dimension of the radius of the wind sector represent the wind speed frequency distribution function [61]. The core of each plot denotes a wind speed of zero, which expands outwardly. As depicted in Figure 3, it is observed that the wind direction is widely distributed, with the west to southwest and the east to northeast wind directions dominating. Strong winds that are above 4.0 m/s are present in all the sectors. Alghamdi et al. [46] reported that wind speed was positively correlated with bioaerosol-bound PM but negatively correlated with the size of PM_2.5_. Wind speed is crucial to the survival of airborne microbes. It acts as a dilution factor, through diffusion, by reducing the net concentration of bioaerosols during transportation from the source to sampling point.

### 3.2. Bacterial and Fungal Concentrations Associated with PM_2.5_

**Figure 4:**
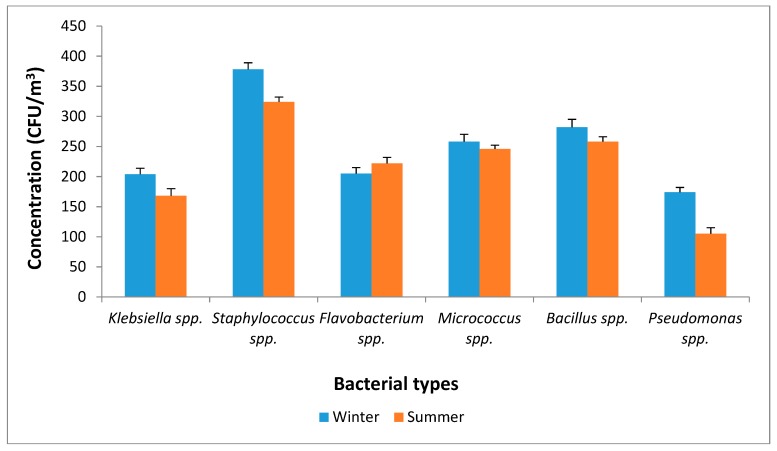
Concentration of isolated bacterial-bound PM_2.5_ in Pretoria West.

A higher concentration of bacteria (168–378 CFU/m^3^) than fungi (58−155 CFU/m^3^) was recorded. Lighthart [22] and Alghamdi et al. [46] reported a higher concentration of airborne bacteria than fungi in PM. The reduced concentration of fungi recorded in our study may be a result of the characteristics of the study area. This can be attributed to the lack of biotic sources and arid and barren terrains. Instances of a significant decrease in the levels of airborne fungi in hot weather conditions have been reported [46,62]. Other factors that can predict low biological-bound PM include meteorological factors, the composition of the PM, physical and chemical changes, the prevailing air pollution and geographical characteristics [63]. High concentration of toxic chemical compounds in PM_2.5_ could cause a reduction in its microbial concentration [63]. Figure 4 shows the types and concentration of bacteria and fungi associated with PM_2.5_. The outdoor airborne bacteria identified in summer were similar to the bacteria found in winter: *Staphylococcus* spp. had the highest concentration, followed by *Bacillus* spp. Other bacteria included *Micrococcus* spp., *Flavobacterium* spp.*, Klebsiella* spp. and *Pseudomonas* spp.

However, *Pseudomonas* spp. was not detected in any of the samples during the winter season. This implies that the concentration of Gram-positive bacteria (*Staphylococcus, Bacillus, Micrococcus*) in urban air is higher than Gram-negative bacteria (*Flavobacterium, Klebsiella, Pseudomonas*). This position has been reported by Fang et al. [64] in Beijing, China and by Aydogdu et al. [65] in Edirne, Turkey. The ability of Gram-positive bacteria to survive under the harsh conditions of intense solar radiation, dryness and aerosolized chemical pollutants enables their concentration in the atmosphere to exceed that of Gram-negative bacteria [64].

**Figure 5:**
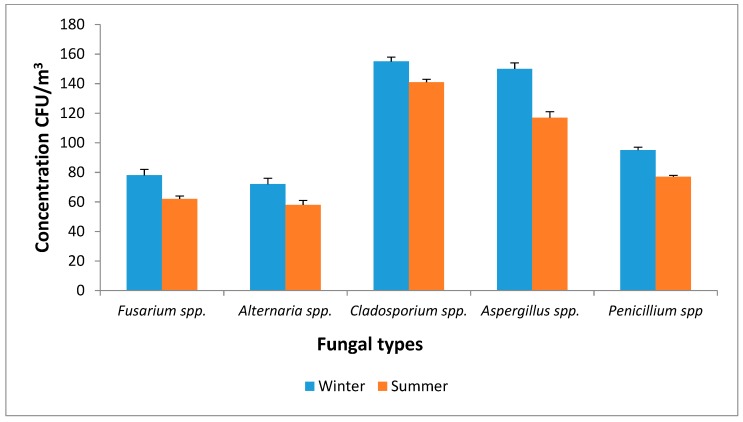
Concentration of isolated fungal-bound PM_2.5_ in Pretoria West

Moreover, the fungal spores identified to be associated with PM_2.5_ in this study include *Cladosporium* spp., *Aspergillus* spp., *Penicillium* spp., *Fusarium* spp. and *Alternaria* spp. Mentese et al., [66] reported that *Alternaria, Aspergillus, Cladosporium,* *Fusarium* and *Penicillium* spores are the prevalent allergic genera. The concentration of *Cladosporium* spp. in winter and summer (155 CFU/m^3^ vs. 141 CFU/m^3^) was higher than that of *Aspergillus* spp. (150 CFU/m^3^ vs. 117 CFU/m^3^), *Penicillium* spp. (95 CFU/m3 vs. 77 CFU/m^3^), *Fusarium* spp. (78 CFU/m3 vs. 62 CFU/m^3^) and *Alternaria* spp. (72 CFU/m^3^ vs. 58 CFU/m^3^) (Figure 5). *Cladosporium* spp. and *Alternaria* spp. are known to be outdoor fungi [67].

This study is consistent with other studies that reported the dominance of *Cladosporium, Penicillium, Aspergillus, Alternaria,* yeasts and non-sporing isolates in outdoor air [68,69]. Of these types, *Cladosporium* spp. were more prevalent than the other fungal types that are associated with PM [46,69]. The abundance of *Aspergillus, Penicillium* and *Alternaria* in PM was credited with their ability to grow on various substrates and under diverse weather conditions. Also, their prevalence is dependent on their ability to produce and discharge high spore numbers into the atmosphere [70]. *Aspergillus* *and* *Penicillium* species are recognized producers of mycotoxins which are injurious to human health at elevated levels [71]. Hypersensitivity to *Aspergillus fumigatus* and *Penicillium* can cause allergic bronchopulmonary, while the persistence and severity of asthma have been linked to human sensitivity to *Alternaria* and *Cladosporium* [72].

Although it may be difficult to associate the concentrations of bacterial and fungal types identified in this study with adverse health outcomes, past studies have pointed out the role of these microorganisms in disease causation. *Aspergillus* spp., *Penicillium* spp., *Alternaria* spp. and *Cladosporium* spp. can induce respiratory conditions such as asthma, allergic rhinitis and hypersensitivity reactions in susceptible individuals [73,74].

Moreover, the lowest bacterial and fungal counts in this study were identified in winter, with the counts being higher in summer. Winter season is synonymous with low ambient temperatures, which in turn affect microbial growth [75]. Low temperatures are not favorable to the growth, reproduction and distribution of microorganisms [76]. A reduced temperature slows down enzymatic action and affects the fluidity of the microbial cell membrane [75]. Moreover, the increased temperatures that are prevalent in summer support the growth and physiological activities of bioaerosols [75].

However, in contrast to the findings of this study, the incidence of higher bacterial and fungal counts in winter than in summer was reported by Li et al. [77] in Northwest China and by Gao et al. [78] in Beijing, China. Researchers are of the view that the survival of bioaerosols in the atmosphere could be hindered by intense solar or ultraviolet radiation exposure that depicts the summer months and hence, the reduction in the abundance of bacterial and fungal counts [79,80].

### 3.3. Dose Rate Estimation

The computed results for the inhaled dose rates of bacteria and fungi in PM_2.5_ are shown in Table 2. It was observed that children (2–5 years) inhaled a higher dose of bacterial and fungal aerosols in winter and summer than other age groups. Overall, children inhaled significantly higher bacterial and fungal doses than infants and adults. This position has been reported in similar studies [51,81,82]. Their higher respiration rates per unit body weight in addition to other behavioral characteristics and physiological features could possibly explain the higher rates among children [83].

Adverse health outcomes in humans are strongly dependent on the level of absorbed dose [74]. Children are more susceptible to the effects of airborne pollution than adults due to their increased ventilation rates, underdeveloped or immature lungs and increased physical activity [84,85]. The constituents of airborne pollutants have the ability to interfere with the signaling pathways of a timed sequence of chemical messages that guides lung growth [86]. Researchers have reported that particulate matter exposure affects the functioning and growth of the lungs in children [87,88]. They possess an airway epithelium that is permeable to inhaled pollutants [89]. Their poor immunity to PM and exposure to outdoor PM has been linked with increased incidence of Lower Respiratory Tract Infections (LRTIs) [90]. The observed health risks in humans from microbial exposure is dependent on the types and concentration of species, metabolic products, exposure duration and susceptibility of individuals [57]. Daily inhalation exposure to low doses of aeroallergens could also weaken mucociliary clearance and the immune system, thus increasing susceptibility to respiratory problems [70].

This study was limited by the culture-dependent method that was adopted. It has been reported that culture-based methods are laborious and can only support the growth of ~10% of the total microorganisms in an environment [91]. The method assumes that organisms will grow and produce typical characteristics within a specified period. Conversely, culture-dependent methods have been adopted previously in bioaerosols studies [92,93,94]. It has been used extensively in the collection and identification of microbial diversity [95] and to reflect real-time changes in the types and concentrations of airborne bioaerosols together with changes in environmental conditions [96,97].

## 4. Conclusions

This study gave an insight into the seasonal concentrations of bacterial and fungal types present in PM_2.5_ and their corresponding inhalation doses in Pretoria West industrial area. Bacterial-bound PM_2.5_ identified in this study included species of *Staphylococcus, Bacillus, Micrococcus*, *Flavobacterium*, *Klebsiella* and *Pseudomonas*. Allergenic and pathogenic fungi that were identified included species of *Alternaria, Aspergillus, Cladosporium*, *Fusarium* and *Penicillium*. The concentrations of both bacteria and fungi were lower in winter than in summer. Children are more likely to inhale significantly higher bacterial and fungal doses than infants and adults.

Findings from this study could serve as the basis for other bioaerosols studies looking at the role of the biological fractions of PM_2.5_ on human health. The information generated could also be used by policymakers and relevant stakeholders in the establishment of regulatory standards pertaining to bioaerosols in the outdoor air. This will assist in the development of an appropriate intervention that will ensure the protection of vulnerable populations from the adverse health effects of exposure to bioaerosol-bound PM_2.5_.

## Figures and Tables

**Table 1:** Recommended values in equations of the daily exposure dose of PM_2.5._

Parameter	Definition	Value for Age Categories				Reference
		Infant (0–1 year)	Child (2–5 years)	Child (6–12 years)	Adult (19–75 years)	
C	Mean concentration of PM_2.5_ in ambient air (μg/m^3^)					
ET	Exposure time (h)	1	8	6	3	[59,60]
InhR	Inhalation rate (m^3^/day)	9.2	16.74	21.02	21.4	[60]

**Table 2:** Age-specific dose rates (CFU/kg/day) of bioaerosols in PM_2.5._

Season	Dose Rate of Bacterial Aerosols				Dose Rate of Fungal Aerosols			
	Infant (0–1 year)	Child (2–5 years)	Child (6–12 years)	Adult (19–75 years)	Infant (0–1 year)	Child (2–5 years)	Child (6–12 years)	Adult (19–75 years)
Winter	210.1	1528.8	718.3	230.7	49.7	361.5	169.8	54.5
Summer	233.7	1700.7	799.0	256.6	63.5	462.2	217.2	69.7
